# Advances in Atmospheric Cold Plasma Technology for Plant-Based Food Safety, Functionality, and Quality Implications

**DOI:** 10.3390/foods14172999

**Published:** 2025-08-27

**Authors:** Siyao Liu, Danni Yang, Jiangqi Huang, Huiling Huang, Jinyuan Sun, Zhen Yang, Chenguang Zhou

**Affiliations:** 1 School of Pharmacy, Jiangsu University, Zhenjiang 212013, China; 2 School of Food and Biological Engineering, Jiangsu University, Zhenjiang 212013, China; 3 Key Laboratory of Geriatric Nutrition and Health, Beijing Technology and Business University, Ministry of Education, Beijing 100048, China; 4 Key Laboratory of Nuclear Agricultural Sciences of Ministry of Agriculture and Zhejiang Province, Institute of Nuclear Agricultural Sciences, Zhejiang University, Hangzhou 310058, China

**Keywords:** atmospheric cold plasma, food safety, mechanisms, functionality, quality

## Abstract

Growing global concerns over pesticide residues and microbial contamination in plant-derived foods have intensified the demand for sustainable decontamination solutions. Conventional physical, chemical, and biological methods are hampered by inherent limitations, including operational inefficiency, secondary pollution risks, and nutritional degradation. Atmospheric cold plasma (ACP) has emerged as a promising non-thermal technology to address these challenges at near-ambient temperatures, leveraging the generation of highly reactive oxygen/nitrogen species (RONS), ultraviolet radiation, and ozone. This review comprehensively examines fundamental ACP mechanisms, discharge configurations, and their applications within plant-based food safety systems. It critically evaluates recent advancements in inactivating microorganisms, degrading mycotoxins and pesticides, and modulating enzymatic activity, while also exploring emerging applications in bioactive compound extraction, drying enhancement, and seed germination promotion. Crucially, the impact of ACP on the quality attributes of plant-based foods is summarized. Treatment parameters can alter physicochemical properties covering color, texture, flavor, acidity, and water activity as well as nutritional constituents such as antioxidants, proteins, lipids, and carbohydrate content. As an environmentally friendly, low-energy-consumption technology with high reactivity, ACP offers transformative potential for enhancing food safety, preserving quality, and fostering sustainable agricultural systems.

## 1. Introduction

Global population growth, accelerated globalization, increased industrial chains, and diversified technological approaches have heightened concerns on the safety of plant-based food. In recent years, frequent incidents involving pesticide misuse, microbial contamination, and excessive heavy metals in plant-derived foods pose significant threats to human health. Pesticide and microbial contamination are particularly concerning due to their potential carcinogenicity, teratogenicity, and mutagenicity [1,2]. Consequently, regulatory oversight in the fields of food safety protection has been progressively intensified within the agricultural and food sectors [3,4,5,6,7]. Current degradation approaches comprise physical (e.g., irradiation, photocatalysis, ultrasound, and microwave [8,9,10]), chemical (e.g., persulfate, Fenton reaction, and ozonation [11,12,13]), and biological methods (e.g., microbial and enzymatic degradation [14,15,16]). However, traditional physical methods tend to be inefficient, while chemical and biological methods may lead to secondary pollution and potential losses in the quality and nutritional value of food or medicinal materials [17]. It is important to note that these conventional methods remain indispensable for many large-scale industrial processes where their efficacy and cost-effectiveness are well established. The search for alternative or complementary technologies is driven by the need to address the specific limitations of these methods, particularly for heat-sensitive, high-value, and minimally processed products where quality preservation is paramount. In contrast, non-thermal technologies such as ultra-high pressure, ultraviolet radiation, pulsed electric fields, and low-temperature plasma represent emerging research frontiers for degrading food contaminants [18,19,20,21,22,23].

Atmospheric cold plasma (ACP), a novel green non-thermal processing technology, demonstrates significant potential in plant-based food processing through the generation of reactive oxygen/nitrogen species, ultraviolet radiation, and ozone. This enables sterilization, pesticide degradation, and enzyme inactivation in plant-derived foods while minimizing thermal damage to nutritional and sensory properties [24]. ACP technology features low energy consumption, ease of operation, high reactivity, thermodynamic non-equilibrium and high efficiency. It selectively targets surface microorganisms or enzymes without sample penetration, thereby maximally preserving nutritional content and natural flavor while leaving no toxic residues. Its operational efficiency supports applications in agriculture and food processing, including sterilization, seed treatment, pesticide degradation, food preservation, surface modification, active compound extraction, and food drying [24].

This review delivers a comprehensive analysis of recent advances in ACP technology for plant-based foods. While several reviews on this topic exist, this work distinguishes itself by three key contributions: first, by systematically delineating ACP generation systems for food-specific applications; second, by moving beyond a descriptive summary to critically analyze and interpret the conflicting findings in the literature regarding ACP’s impact on food quality; and third, by identifying the causal relationships between processing parameters, food matrix properties, and observed outcomes. By synthesizing these perspectives, we aim to provide a forward-looking perspective on the future directions of ACP applications.

## 2. Types of Atmospheric Cold Plasma

**Figure 1:**
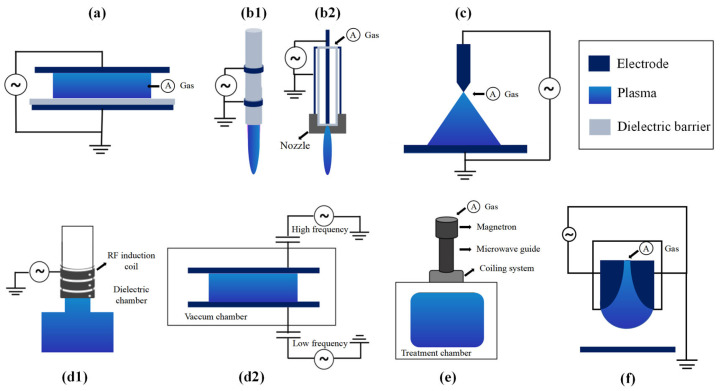
Examples of various plasma source devices [31,32,33] (reprinted with permission from ref. [32], copyright © 2025 Springer Nature): (**a**) DBD, (**b1**) double-ring electrode DBD jet, (**b2**) coaxial electrode APPJ, (**c**) CD, (**d1**) inductively coupled plasma (ICP), and (**d2**) capacitively coupled plasmas (CCP), (**e**) MWD source, and (**f**) GAD.

Plasma is the fourth state of matter. It was identified during the 17th century and formally termed by Irving Langmuir in 1928 [25,26]. This ionized neutral gas comprises excited electrons, ions, radicals, and neutral particles [27]. While all gases above zero Kelvin contain charged particles, plasma requires collective particle interactions. Generated via energy inputs, including electrical, UV, and gamma radiation, plasma emits 100–380 nm UV radiation alongside highly reactive species [28]. Classification depends on gas temperature and thermodynamic equilibrium: high-temperature plasma contrasts with low-temperature variants—thermal plasma and cold plasma. ACP specifically denotes atmospheric-pressure cold plasma where ions or neutrals exhibit lower temperatures than electrons, creating a non-uniform energy distribution [28,29]. ACP types include dielectric barrier discharge (DBD), atmospheric pressure plasma jet (APPJ), corona discharge (CD), radio frequency (RF), gliding arc (GAD), and microwave discharge (MW), with source structure and reactive species density determining the application efficacy [30]. Various plasma source devices are illustrated in Figure 1, and the comparative analysis of major ACP generation systems is summarized in Table S1.

### 2.1. Dielectric Barrier Discharge

Dielectric barrier discharge (DBD), a prevalent ACP generation method, employs two metal electrodes, one connected to a high voltage source and the other grounded. At least one of the electrodes is covered with a dielectric layer (such as ceramic, glass, quartz, or polymer), or a dielectric barrier is suspended between the electrodes. This configuration prevents arc or spark discharges while maintaining a millimeter-scale gas gap filled with air, nitrogen, argon, or mixtures [34]. Operated at atmospheric pressure with alternating current or pulsed voltages typically at kilohertz to megahertz frequencies (1–500 kHz), and in special cases from 50 Hz to 10 MHz [35], DBD initiates when electrons in the gas gap are accelerated by the applied electric field. Their collisions with neutral molecules trigger localized ionization, forming spatially discrete filamentary discharges, or under optimized conditions uniform glow discharges. The accumulation of surface charges on dielectrics generates a self-pulsing mechanism that quenches individual micro-discharges and prevents arc transition [36]. Processing efficacy depends on parameters (e.g., pressure, gas, type, flow rate, power, and duration), reactor geometry, substrate properties, and exposure conditions. Common DBD configurations include surface discharge (symmetrical electrodes for planar treatment), volume discharge (coaxial annular gap for pollutant degradation), and specialized variants like floating-electrode, flexible-film, or radiofrequency-driven systems [35,36]. Key advantages encompass uniform discharge, low energy consumption, compact design, operational safety, and cost-effectiveness [34].

### 2.2. Corona Discharge

Corona discharge (CD) generates non-thermal plasma through localized electron avalanches occurring within a strongly non-uniform electric field maintained at voltages below the breakdown threshold [34,37]. It occurs between geometrically asymmetric electrodes, typically involving a sharp conductor (e.g., needle or wire) and a significantly larger counter-electrode (plate/cylinder), utilizing DC, AC, or pulsed power sources [38]. Positive CD initiates at the anode tip via photoionization-driven streamers, while negative CD originates at the cathode tip through electron-impact ionization. In both cases, the discharge development is critically constrained by the accumulation of space charge [39]. Discharge morphology evolves with applied voltage and electrode geometry: needle–plate configurations exhibit distinct regimes including pulsed-to-glow transitions (positive) or Trichel pulse-to-spark stages (negative), whereas wire-based systems typically form filamentary and often beaded jets (negative) or uniform sheathed streamers (positive) [38]. CD finds applications in areas such as electrostatic precipitation, ozone generation, and surface sterilization. However, its widespread adoption is often limited by challenges including poor spatial uniformity, risk of localized thermal damage, and relatively small effective treatment areas. These limitations are frequently addressed via multi-point electrode arrays or hybrid plasma systems [31,38,40].

### 2.3. Atmospheric Pressure Plasma Jet

The atmospheric pressure plasma jet (APPJ) generates non-equilibrium plasma beyond its discharge gap, comprising coaxial or ring-shaped electrodes within inert or mixed gases [31]. Applying high-frequency, low-frequency, or nanosecond pulses at 100–250 V ionizes the gas, ejecting ionized molecules as a plume through a nozzle into an unconfined working zone [32]. Gas flow rate and applied voltage critically govern plasma generation in this centimeter-scale AC-powered device [31,41]. APPJs exhibit streamer and glow discharge characteristics [41], with discharge-working zone separation ensuring stability [42] and gas flow promoting uniformity [43]. While inert gases facilitate discharge initiation, gas mixtures are also viable [43]. APPJs eliminate vacuum systems, enabling remote treatment of complex surfaces [41,43] for applications including sterilization, biomedicine, and material processing.

### 2.4. Gliding Arc Discharge

Gliding arc discharge (GAD) generates non-thermal plasma by initiating high-voltage arcs at the narrowest electrode gap, which are elongated by gas-dynamic shear forces and glide along electrodes under gas flow or mechanical motion [44]. This cyclic process involves local breakdown, arc elongation driven by flow velocity gradients, and re-ignition after arc extinction at maximum length due to resistance-induced power imbalance [45]. GAD systems utilize conductive metal electrodes to control gas flow profiles, powered by DC/AC high-voltage sources [44]. Rotary designs overcome planar GAD’s gas interaction limitations by confining rotating arcs in vortex-stabilized zones [45]. Compared to other plasmas, GAD achieves high ionization rates, gas temperatures in the arc core to 300–1000 K in downstream regions, and improved energy efficiencies in optimized rotary configurations [45]. Its low energy consumption, rapid quenching that preserves non-equilibrium states, and chemical selectivity [44] support applications in fuel conversion, material synthesis, and pollution control [46].

### 2.5. Glow Discharge

Glow discharge (GD), a self-sustained low-pressure (1–1000 Pa) plasma generated between parallel electrodes via an external electric field, produces characteristic glow emission through electron-impact ionization and excitation [47,48]. It operates across DC/AC/RF modes [48], with discharge characteristics governed by voltage, frequency, and gas flow [43]. In DC mode, the discharge exhibits eight alternating bright/dark zones (e.g., Aston dark space, cathode glow, negative glow, positive column) due to the electric field and energy gradients [49]. Typical systems comprise vacuum chambers, electrodes, HV power, and gas/vacuum units. These systems enable surface modification and thin-film deposition that can be applied in biomedicine [50] and environmental remediation [51], with the advantages of low energy consumption, high uniformity, and eco-friendliness [47]. Limitations include vacuum-system complexity and cost [52]. In recent years, in order to expand the application in biomedicine, the research focus has gradually shifted to atmospheric-pressure glow-discharge (APGD) technology.

### 2.6. Radio Frequency Discharge

Radio frequency discharge (RFD) plasma is generated by ionizing gases via high-frequency alternating electric fields, where oscillating electrons accelerated by electromagnetic fields induce ionization through collisions with neutrals [53]. Power coupling efficiency and plasma uniformity depend on the RF excitation design, achieved either by applying RF voltage across parallel electrodes or inducing currents via external coils, optionally isolated by dielectric windows [54]. Typical systems include RF power supplies, reaction chambers with electrodes or coils, gas or vacuum units, and cooling, though primarily low-pressure cold plasma, atmospheric operation is feasible [55,56]. This technology enables low-temperature, high-activity plasma with tunable density or energy distribution, avoiding thermal damage to sensitive materials, but faces challenges in equipment complexity, electromagnetic interference, and shielding requirements. Key applications span semiconductor manufacturing, surface engineering, biomedical uses, food and textile modification [53], and seed germination control [57].

### 2.7. Microwave Discharge

Microwave discharge plasma (MWD) is generated by ionizing gases with high-frequency microwaves (0.3–300 GHz, typically 2.45 GHz) from a magnetron, operable at low or atmospheric pressures [54]. Microwaves delivered via waveguides accelerate gas electrons through elastic collisions, inducing ionization and UV and visible photon emission without electrodes, eliminating filamentary discharge risks [33,58]. Key features of microwave discharge plasma include high electron density, efficient production of reactive oxygen or nitrogen species, and its electrode-free operation, which eliminates contamination risks. Systems comprise magnetrons, resonant chambers, and gas units [58], classified by excitation mode or pulsing [59]. MWD is typically applied in the manufacturing of advanced materials, environmental governance, space propulsion, medical treatment, and other fields [54].

### 2.8. Plasma-Activated Liquid

Plasma-activated liquid (PAL) is generated by exposing liquids to direct or indirect low-temperature plasma [60,61], inducing physicochemical alterations including decreased pH, shifted oxidation–reduction potential, and generation of stable and short-lived reactive species [58]. These changes enable microbial inactivation and organic contaminant degradation [62], with potential metal ion release from electrode erosion [63]. PAL production efficiency depends on plasma–liquid distance, gas type, and treatment conditions [58], achievable via diverse discharges including MWD, DBD, RFD, and GAD. Key advantages comprise cost-effectiveness, elimination of UV-induced damage, enhanced penetration efficacy within microstructures [62], and application scalability. However, a critical challenge for the widespread application of PAL is the stability of its reactive species, which dictates its shelf-life. The activity of PAL is generally maintained for a limited of time (from hours to several days), depending on the liquid’s composition and storage conditions such as temperature and light exposure [62]. Applications span microbial or viral inactivation, pesticide degradation, seed activation, food processing, and nanomaterial synthesis [63]. The antimicrobial and degradation efficacy of PAL is attributed to a complex mixture of short-lived and long-lived reactive species generated in the liquid [64]. The stability of long-lived species is paramount for the “storability” of PAL. Recent research indicates that while physicochemical properties like pH, oxidation–reduction potential (ORP), and electrical conductivity (EC) can remain stable for up to 30 days at 4 °C, the concentrations of key antimicrobial agents like H_2_O_2_ and nitrites can decay significantly after just 5 days of storage. This inherent instability presents a major bottleneck, and future research must focus on developing stable PAL formulations, possibly through the use of additives (e.g., stabilizers, buffers) or optimized generation protocols, to realize its full industrial potential [65].

## 3. ACP Applications for Plant-Based Foods

Cold plasma treatment extends food shelf-life by inactivating hydrolytic enzymes, suppressing microbial growth, decomposing pesticides, and eliminating insects [19]. It also enhances bioactive compound extraction, assists drying of aromatic plants, and promotes seedling germination and growth. This section focuses on ACP applications in plant-derived foods.

### 3.1. Microbial Inactivation

Conventional microbial decontamination of plant-based foods risks promoting antibiotic-resistant pathogens and altering food composition through chemical residues [19]. ACP overcomes these limitations by inactivating broad-spectrum microorganisms including bacteria, fungi, spores, viruses, and biofilms, while preserving food quality through near-ambient temperature operation [36,66,67,68,69,70,71,72,73,74,75]. The effect of ACP treatment on microbial inactivation is presented in Table 1. The antimicrobial efficacy arises from three synergistic mechanisms detailed below.

**Figure 2:**
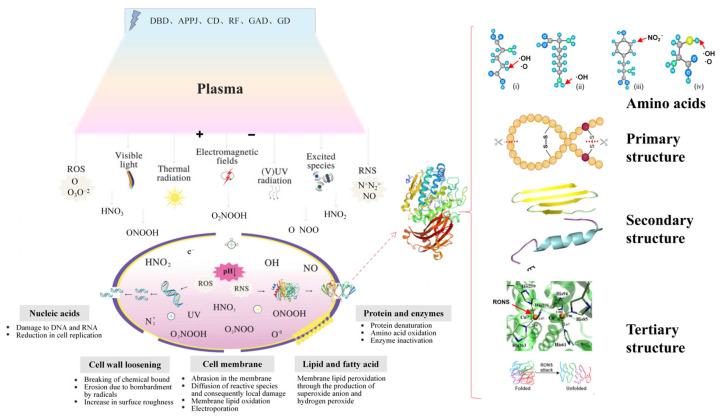
Mechanism of cold plasma affecting microorganism cells [75] and enzyme activity [112] (reprinted with permission, copyright © 1999–2025 John Wiley & Sons).

Reactive species induce structural damage to microbial envelopes. Reactive oxygen or nitrogen species damage microbial envelopes by disrupting chemical bonds in peptidoglycan or lipopolysaccharide layers [71,76,77]. The primary mechanisms of microbial inactivation are illustrated in Figure 2. This process triggers lipid peroxidation and amino acid oxidation in membranes, ultimately causing cell death. For instance, RF plasma treatment inactivated *Escherichia coli* (*E. coli*) on walnuts through membrane rupture [78], while DBD plasma disrupted *Bacillus tequilensis* on black peppercorns [79], and APPJ achieved complete *E. coli* inactivation [80].

Charged particles cause electrostatic membrane breakdown. Electrons and ions generate electrostatic stress exceeding membrane tensile strength, resulting in cytoplasmic leakage and intracellular invasion of ROS/RNS that oxidatively damage DNA [81], inhibiting proliferation [71]. This mechanism is evidenced by APPJ treatment smoothing *Bacillus subtilis* membranes in black pepper and liquid systems [82].

The combined action of the mechanisms allows ACP to eliminate diverse microorganisms: spore-formers like *Bacillus subtilis* [83] and *Bacillus cereus* [84], fungi including yeasts [85] and *Aspergillus bisporus* [86], parasitic protozoa such as *Cryptosporidium parvum* oocysts [87], and pathogenic bacteria such as *Salmonella enterica* [88], *Listeria monocytogenes* [89], and *Candida albicans* [90].

Inactivation efficacy is modulated by experimental parameters. Environmental conditions (pH, moisture, temperature) influence microbial susceptibility [19], while experimental settings (gas composition, voltage, treatment time) govern reactive species generation [19,79]. Microbial characteristics (species, initial load) further determine resistance levels [19]. For example, *Aspergillus* inactivation on walnuts required optimized RF plasma parameters [78], and *Bacillus tequilensis* reduction on black peppercorns correlated with water activity, treatment duration, and voltage [79].

**Table 1:** Effect of ACP treatment on microbial inactivation.

No.	Sample	Microorganism	Equipment	Parameters	Results	Reference
1	*Juglans regia* L.	*Coliforms*, Molds	RF	20, 30, 40, 50 W; 10, 15, 20 min	Microbial activity decreased gradually with increasing power and time	[78]
2	Black peppercorns	Indigenous bacteria, *Bacillus tequilensis* spores	DBD	9.7, 9.8, 10.2, 10.5, 10.6 kV; 7.9, 10.0, 15.0, 20.0, 22.1 min	Microbial inactivation improved with increased water activity, treatment time, and voltage	[79]
3	Black pepper grains	*Bacillus subtilis* vegetative cells and spores	APPJ	280 GHz, 50 mA, 600 W, 30 min	Microbial activity decreased with increased voltage and treatment time	[82]
4	Black pepper seeds, allspice berries, and juniper berries	*Aspergillus niger*, *Bacillus subtilis*	MW	Argon, 20 L/min, 2.45 GHz, 600 W, 15–60 s	Degree of bacterial inactivation increased with prolonged treatment time	[83]
5	Black pepper	*Bacillus cereus*	APPJ	40, 50, 60 L/min; 800, 900, 1000 W; 0–10 min	Significant reduction in vegetative cells of *Bacillus cereus*	[84]
6	*Coriandrum sativum*	*Cryptosporidium parvum* oocysts	APPJ	47 GHz; 549 W; 0, 30, 90, 180 s	Microbial activity decreased with increased treatment time	[87]
7	*Curcuma longa* var. Suvarna	Aerobic viable cell	DBD	25 kV; 3, 5, 7 min	Antibacterial effect declined with prolonged processing time	[91]
8	Dried saffron stigma	Molds, Yeasts, *Escherichia coli*	APPJ	Air/Argon, 1 L/min; 40, 70, 100 W; 1, 5, 10 min	Microbial load decreased with increased exposure time	[92]
9	*Crocus sativus* L.	*Escherichia coli* (*E. coli*)	APPJ	Helium, 1 L/min; 12 GHz; 5, 6, 7 kV; 0–12 min	Complete *E. coli* inactivation	[80]
10	*Crocus sativus* L.	TVC, *Coliforms*, Molds, Yeasts	RF	Oxygen, 70, 90, 110 W; 5, 10, 15, 30 min	Maximum microbial log reduction at 110 W for 30 min	[93]
11	Red mini-roses	Microbiota diversity	DBD, GD	962 Hz, 18 kV, 20 min	Reduced microbial diversity without altering dominant populations	[94]
12	*Agaricus bisporus*	Aerobic colony	DBD	Optimal: 95 kV, 130 Hz, 10 min	Effective spoilage microbe reduction, minimizing contamination risk	[86]
13	*Pleurotus ostreatus*	Soil-borne pathogens	DBD	2 L/min, 6 kV; 5–25 min	Optimal at 25 min; colony count decreased with time	[95]
14	*Flammulina velutipes*	Adherent Bacteria, *Escherichia coli*	APPJ, PAW	83 kHz, 0.68 kV, 77 mA, 0–30 min	CFU reduction proportional to treatment time; NTAPPJ reduced bacterial adhesion; PAW increased cell death and lipid peroxidation	[96]
15	Shiitake mushrooms	Bacteria	DBD, GAD-PAW	N_2_-O_2_ (3:1), 200 W, 1200 W, 20 min	PAW outperformed DBD in postharvest quality preservation	[97]
16	*Vitis vinifera*	Aerobic bacteria, *E. coli*	APPJ	Helium, 20 kHz, 6.5 W, 4–5.5 kV, 1–3 min	Complete aerobic bacteria reduction; extended shelf-life (28 days)	[98]
17	*D. longan*	*Diutina catenulata*	DBD	Argon/air mixtures, 4 L/min, 5 kHz, 53 W, 24 kV, 180–360 s	Reduced microbial density; expanded antibacterial zones over time	[90]
18	Dried jujube	*Aspergillus niger* spores	DBD	50 Hz; 50–70 kV; 0–20 min	Reduced spore viability and toxicity	[99]
19	Palm dates	*Aspergillus niger*	DBD	10 kHz, 8–30 W, 5–10 kV, 3 min	Inhibited *A. niger* growth	[100]
20	Cowpea	*Callosobruchus maculatus*	DBD	65% O_2_/30% N_2_/5% CO_2_; 20–70 kV, 1–3 min	Mortality/sterility increased with time and voltage	[101]
21	*Cichorium intybus* L.	Biofilms of *Pseudomonas aeruginosa*, *E. coli*	APPJ	Helium, 2 SLM, 25 kHz, 8 kV, 3 min	Membrane disruption; *E. coli* more sensitive than *P. aeruginosa*	[102]
22	*Camellia sinensis* var. *sinensis*	Molds, yeasts, *E. coli*, *Enterococcus faecalis*	DBD	20–25 kV; 2–8 min	Complete inactivation at 25 kV/8 min; *E. coli* required higher MIC/MBC than *S. aureu*	[103]
23	Almond slices	Molds, yeasts, *Staphylococcus aureus*	APPJ	Helium, 10 SLM, 17 V, 2.26 A, 5–20 min	Microbial reduction proportional to treatment duration after 20 min	[104]
24	Mulberries	*E. coli*	DBD	0.1–1 A, 30 s	ROS accumulation induced apoptosis; damage intensity current-dependent	[105]
25	Vegetables/fruits/nuts/ powders	*E. coli* O157:H7, *S. Typhimurium*, *Listeria monocytogenes*	DBD	14.4 kHz, 51.7 W, 8 kV, 30 min	Surface roughness negatively correlated with inactivation efficiency	[106]

### 3.2. Enzyme Activity

In plant-based food systems, endogenous enzymes significantly influence physicochemical quality attributes including textural properties, color characteristics, flavor profile, and storage stability [107]. Certain enzymes, such as pectinases and esterases, play a positive role in biotransformation processes such as flavor development and postharvest ripening. However, lipolytic enzymes and oxidoreductases may lead to undesirable effects, including nutrient loss, accelerated browning, and off-flavor formation [19]. ACP has emerged as a non-thermal alternative to conventional processing and has attracted extensive research into its effects on enzyme activity [108].

Plasma treatment systematically alters enzyme structures. The mechanism of cold plasma affecting microorganism cells and enzyme activity is shown in Figure 2. For instance, DBD exposure treatment at varying durations reduced peroxidase (POD) and horseradish peroxidase (HRP) activity via conformational shifts involving decreased α-helix and increased β-sheet content, which exposes hydrophobic aromatic amino acids and heme groups [109]. Jia et al. [110] reported that plasma treatment under optimized conditions suppressed chlorophyllase and pheophorbide a oxygenase (PAO) activity in tomatoes while downregulating the expression of chlorophyll metabolism-related genes, thereby delaying chlorophyll degradation. Subsequent studies confirmed inhibition of enzymes during storage, including polygalacturonase (PG), pectin methylesterase (PME), 3α-L-arabinofuranosidase (3α-L-Af), 4β-galactosidase (4β-Gal), cellulase (Cx), and β-glucosidase (β-Glu) [111].

ACP regulates enzyme activity through dynamic multifactorial interactions at physical, chemical, and biological levels [113], influenced by treatment conditions, plasma source, and processing volume. Inactivation or activation depends on enzyme-reactive species contact degree, enzyme stability, structural integrity, and stress response capacity [114]. Enzymes categorize as ACP-resistant such as lipases, catalase, and superoxide dismutase, or ACP-sensitive such as phenol oxidases, peroxidases, and lysozymes. Resistant enzymes show transient activity enhancement under mild oxidative stress via conformational optimization or active site exposure, declining with prolonged exposure. Sensitive enzymes rapidly succumb to ROS/RNS, which attack critical groups and induce denaturation/peptide bond cleavage/aggregation [19]. Amino acid residue can be modified and its secondary structure changed during ACP treatment. Amino acids undergo oxidative, sulfonylation, hydroxylation, amidation, or ring-opening reactions with ROS or radicals, particularly at side chains like thiol groups and aromatic rings, where sulfhydryl residues form disulfide bonds promoting aggregation [113]. Additionally, ACP targets secondary structures, significantly reducing α-helix while increasing β-sheet content in polyphenol oxidase (PPO) and peroxidase (POD) to suppress browning-related enzyme activity [114,115].

### 3.3. Mycotoxin Degradation

Mycotoxins such as aflatoxin B1 (AFB1), ochratoxin A (OTA), and deoxynivalenol (DON) are toxic fungal metabolites prevalent in plant-based foods, posing significant health risks [116,117,118,119,120]. ACP degrades these contaminants through three primary mechanisms: direct molecular decomposition, inactivation of toxigenic fungi or spores, and interference with biosynthesis pathways [121].

**Figure 3:**
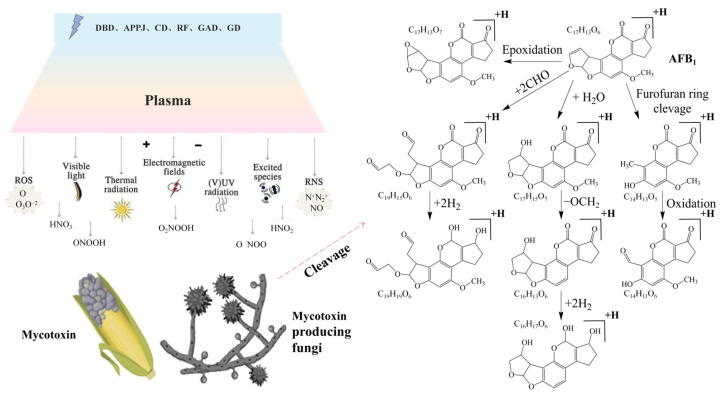
Cold plasma technique in controlling mycotoxins [29] (reprinted with permission, copyright © 2025 Elsevier B.V.) and degradation pathways of aflatoxin (AFB1) [30,122,127,128] (reprinted with permission from ref. [30], copyright © 2025 Informa UK Limited).

ACP directly fragments mycotoxin molecules through radicals and reactive species generated during treatment [122]. The degradation efficiency depends on molecular structure, with key functional groups targeted by reactive oxygen species (ROS) and ultraviolet radiation. Taking AFB1 as an example (Figure 3), radicals (•OH, H•) and ROS (O_3_, H_2_O_2_) oxidize critical structures, including the C8=C9 double bond, lactone ring, and cyclopentenone group responsible for its carcinogenicity [30]. Similarly, DON’s toxicity stems from the C3 hydroxyl, C9=C10 double bond, and C9-C10 epoxy groups [121], while patulin’s conjugated double bonds and lactone ring are vulnerable [121].

Degradation efficacy is influenced by food matrix complexity. Mycotoxins embedded in plant components degrade slower than in pure solutions due to interactions with proteins or carbohydrates. Zhang et al. [123] demonstrated this effect where DON degradation reached 98% in aqueous solution but only 61% in wheat grain.

Synergistic effects enhance detoxification. While UV radiation from ACP alone is insufficient for complete degradation [122], its combination with radicals amplifies fragmentation. Increased H_2_O_2_ and NO_3_^−^ concentrations in plasma-treated cells confirm radical involvement [124]. Akhavan-Mahdavi et al. [125] validated this synergy, showing DBD plasma simultaneously reduced aflatoxin levels and mold/yeast counts in pistachios without altering color, moisture, hardness, or sensory properties. Esmaeili et al. [126] further confirmed no significant changes in free fatty acids or polyphenols after optimized plasma treatment. The effect of ACP treatment on mycotoxin degradation is shown in Table 2.

However, degradation products require careful assessment. Although fragmentation typically yields non-toxic compounds (e.g., formic acid, CO_2_), some metabolites may retain or increase toxicity [30]. Therefore, systematic evaluation of detoxification efficacy across mycotoxin classes remains essential [30].

**Table 2:** Effect of ACP treatment on mycotoxin degradation.

No.	Sample	Mycotoxin	Equipment	Parameters	Results	Reference
1	*Pistacia vera* L.	Aflatoxin	DBD	Oxygen, 25 kHz, 89 W, 15 kV, 60/120 s	Significantly reduced the number of molds and yeasts after 120 days of storage; notably decreased aflatoxin content	[125]
2	*Pistacia vera* L.	*Aspergillus flavus*, Aflatoxin	DBD-APPJ	Air–argon (0%, 50%, 100%), 10–20 kV, 5–15 min	High degradation rate under optimal treatment conditions; no significant difference between control and cold plasma-treated samples	[126]
3	*Pistacia vera* L.	Aflatoxin B1 (AFB1)	RF	12.56 kHz, 80 W; 10, 15 min	Aflatoxin content significantly decreased	[129]
4	Wheat	Deoxynivalenol (DON)	DDBD	160–240 Hz, 60–140 V, duty cycles 20–99%, 25 min	ACP achieved a degradation efficiency of up to 98% in aqueous DON solutions, while 61% degradation in wheat samples	[123]
5	Corn kernels	Aflatoxin B1 (AFB1)	DBD	0.18 W/cm–0.31 W/cm, 30–480 s	Complete removal of aflatoxin B1 (AFB1)	[130]

### 3.4. Pesticide Degradation

**Figure 4:**
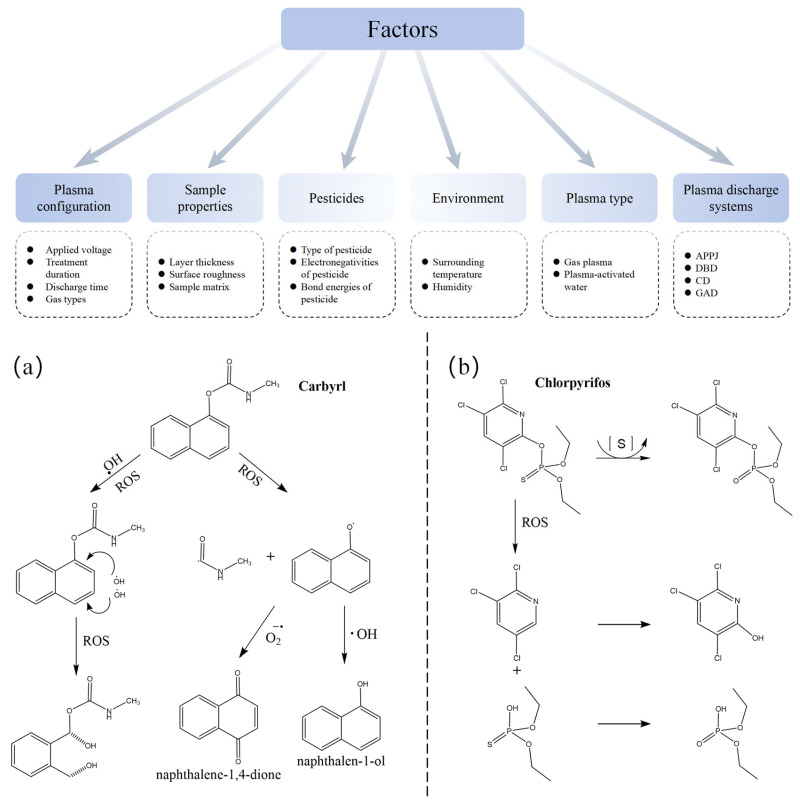
Effect of pesticide degradation factors [31] and proposed degradation pathway of (**a**) Carbyrl and (**b**) Chlorpyrifos [62] (reprinted with permission, copyright © 2025 Springer Nature).

Pesticides, chemical agents that are crucial in agricultural production, have become primary contaminants in plant-based foods due to large-scale application [8,131], posing risks of acute or chronic poisoning, cancer, reproductive disorders, immune dysfunction, and neurological damage in humans [132]. Effective methods for eliminating pesticide residues remain key to reducing human exposure risks [133,134,135]. Cold plasma demonstrates superior pesticide degradation efficacy and has attracted significant attention recently [136]. Facilitating practical applications requires understanding mechanisms and detecting intermediates [131]. ACP primarily degrades pesticides through synergistic physical–chemical effects, as illustrated in Figure 4. The effect of ACP treatment on pesticide degradation is summarized in Table 3. The core mechanisms involve several key processes.

First, degradation is predominantly driven by highly reactive oxygen and nitrogen species (ROS/RNS), with the potent hydroxyl radical (•OH) playing a particularly crucial role [31]. The specific action of •OH varies with pesticide structure. For organophosphates such as malathion, parathion, or chlorpyrifos, •OH radicals target the sulfur atom in the thiophosphate group (P=S), converting it to a more labile P=O structure. This transformation is often followed by cleavage of vulnerable P-S or C-S ester bonds, fragmenting the molecule into smaller, less toxic intermediates that are subsequently more readily mineralized. In contrast, for pesticides containing stable aromatic rings, such as atrazine or carbaryl, •OH radicals initiate hydroxylation reactions and subsequent ring-opening. This disrupts the stable ring structure, leading to carboxylic acid formation and eventual mineralization into CO_2_, H_2_O, and inorganic ions.

Second, concurrently generated ultraviolet (UV) radiation (100–380 nm) during plasma discharge contributes significantly. This UV radiation possesses sufficient energy to directly cleave chemical bonds within pesticide molecules, a photolytic effect that works synergistically with the chemical attacks initiated by the reactive species [137].

Third, the high-voltage electric fields inherent within the plasma exert an influence. These fields can induce molecular polarization or ionization of pesticide molecules, weakening their chemical bonds. This bond weakening renders the molecules more susceptible to attack by ROS/RNS, thereby promoting more efficient degradation overall.

Finally, the degradation pathways are sensitive to the system pH, exhibiting distinct mechanisms under different conditions. Under acidic conditions, hydroxyl radicals generated via water ionization directly oxidize pesticides. Conversely, in alkaline environments, hydroxyl radicals recombine to form hydrogen peroxide, making indirect oxidation the more dominant degradation process [31,138].

**Table 3:** Effect of ACP treatment on pesticide degradation.

No.	Sample	Pesticide	Equipment	Parameters	Results	Reference
1	Fresh *Spinacia oleracea* L.	Chlorpyrifos, Malathion	Volume DBD	100 W, 20 kV, 20 min	Microbial contamination and pesticide residue analysis showed that cold plasma treatment significantly reduced spinach contamination; chlorpyrifos and malathion decreased by 90%	[139]
2	*Glycine. max* (L.) Merr.	Chlorpyrifos	DBD	1–2 kV, 2–6 min	Cold plasma (CP) treatment achieved a degradation rate of 65% for pesticides on soybeans, even high concentrations degraded by 50%	[140]
3	Blueberry	Boscalid, Imidacloprid	DBD	60/80 kV; 2, 5 min	Degradation rates: Boscalid 80%, Imidacloprid 76%; after 1 min of cold plasma treatment, polyphenol and flavonoid content increased	[141]
4	Grape, Strawberry	Chlorpyrifos, Carbaryl	PAW	1 kHz, 5.66 W, 5–30 min	Chlorpyrifos reduction: grape 79%, strawberry 69%; carbaryl reduction: grape 86%, strawberry 73%; no significant changes in color and firmness; slight changes in ascorbic acid levels	[62]
5	Lettuces	Chlorpyrifos, Malathion	DBD	60–80 kV, 30–180 s	DBD treatment significantly degraded malathion and chlorpyrifos in water and lettuce; at 80 kV for 180 s, degradation efficiencies were 64.6% and 62.7%, respectively; no noticeable damage to lettuce quality including color and chlorophyll content; ascorbic acid significantly decreased	[142]
6	Corn	Chlorpyrifos, Carbaryl	DBD	100–1200 Hz, 150–1500 mL·min^−1^, 4–20 W, 20–60 s	Under 1000 mL·min^−1^, 20 W, 1200 Hz for 60 s, chlorpyrifos degradation efficiency reached 86.2%, Carbaryl 66.6%; moisture and starch content significantly decreased, acid value increased, vitamin B_2_ unchanged	[143]
7	Edible Wolfberry	Omethoate	Surface DBD	9 kHz, 0–20 kV, 0.1–30 min	At 10 kV for 30 min, optimal degradation rate reached 99%, with complete conversion into non-toxic species (e.g., PO_4_^3−^, H_2_O, SO_4_^2−^, CO_2_)	[144]
8	*Lycium barbarum*	Omethoate, Dichlorvos (DDVP)	Gas Phase Surface Discharge Plasma (GPSD)	5–15 kV, 0.5–30 min	Maximum degradation rates reached 99.55% and 96.83%; completely degraded into non-toxic species with no impact on *Lycium barbarum* quality	[145]
9	*Solanum lycopersicum*	Chlorothalonil	PAL-U (PAW, PABS)	600 W, 7 kV, 20 L/min; solution treatment 1–10 min, soaking for 15 min	Maximum reduction in residue was 89.23%, with no impact on sample quality	[146]
10	*Solanum lycopersicum*	Chlorothalonil (CTL), Thiram (THM)	PAW, Plasma activated buffer solution (PABS)	Argon/Oxygen = 98/2, 600 W, 2–7 kV, 1–10 min	Degradation rates: 85.3% and 74.2%, respectively; oxidation–reduction potential (ORP) and electrical conductivity (EC) of the solution significantly increased; pH decreased with activation time; no significant effect on tomatoes	[147]

### 3.5. Bioactive Compound Extraction

Plant-derived bioactive compounds exhibit beneficial effects on human health and demonstrate potential in reducing certain diseases [148,149,150,151,152]. Cold plasma emerges as a green extraction alternative to traditional methods [153,154,155,156], offering low environmental pollution, cost-effectiveness, high extraction efficiency, low energy consumption, and reduced water usage [28]. Under mild conditions, ACP enhances bioactive extraction while yielding extracts with favorable antioxidant and antimicrobial activities [26]. Rezaei et al. [157] reported ACP treatment significantly improved various physicochemical properties of extracted oils while increasing protein content. Zhou et al. [158] demonstrated reduced extraction time and solvent consumption of *Lonicera caerulea* with both extraction efficiency of active components and antioxidant activity notably enhanced. Elmizadeh et al. [159] achieved higher tanshinone yield from *Salvia subg. Perovskia root* via combining ACP with microwave extraction.

ACP-generated electrons, ions, and ROS/RNS create cell wall micropores or fractures through oxidation and etching effects to facilitate bioactive release. Additionally, reactive species oxidatively modify cell wall components such as pectin/cellulose/lignin, reducing structural stability and enhancing solvent permeability [160]. Concurrently, reactive species-mediated oxidation improves target compound solubility, hydrophilicity, and other physical properties. Furthermore, UV photons may activate specific enzymes such as phenylalanine ammonia-lyase, or upregulate genes to boost phenolic extraction [28]. ACP effectively disrupts specialized surface structures like glandular trichomes, which are key sites for essential oil biosynthesis and storage. Moderate treatment enhances oil extraction, whereas excessive treatment may cause trichome rupture, reducing yield and altering oil composition [26,161]. For DBD-treated *Mentha spicata* L., studies have shown that elevating power increased essential oil (EO) yield with higher oxygenated monoterpenes but lower monoterpene hydrocarbons, when compared to controls [162]. DBD pretreatment boosted bioactive components with enhanced radical-scavenging capacity [163], and promoted phenolic release via modifying clove morphology [164]. Additionally, studies confirmed plasma technology’s efficacy in enhancing extraction yields for soybean protein [165] and galactomannan [166], alongside improving water absorption and other physicochemical properties.

ACP remains laboratory-scale, with efficiency depending on plasma parameters, gas type, plant species, and bioactive composition. For instance, nitrogen plasma yields higher total phenolic content (TPC) than other gases. Lu et al. [167] demonstrated nitrogen-derived reactive species activated seed bioactives, whereas air plasma species suppressed them. Further optimization requires elucidating plant–plasma interactions and preventing oxidative byproduct formation [26,28].

### 3.6. Drying

The drying process critically extends shelf-life, and influences the quality of plant-based foods [112,168], but conventional techniques show some limits, such as inefficiency, prolonged duration, quality deterioration, uneven dehydration, and high costs [169,170,171,172,173,174,175]. Cold plasma pretreatment enhances drying performance by reducing energy consumption, maintaining product quality, and degrading pathogenic microorganisms [112,176,177,178,179]. Kamkari et al. [180] demonstrated air-DBD plasma treatment doubled drying rate with 66% higher moisture diffusivity, 24% lower energy, and 18% decreased shrinkage in cumin. Similarly, ACP improved the *Lycium barbarum* rehydration ratio via structural modifications facilitating intracellular water and phytochemical release, thereby promoting cellular contraction during drying [181].

**Figure 5:**
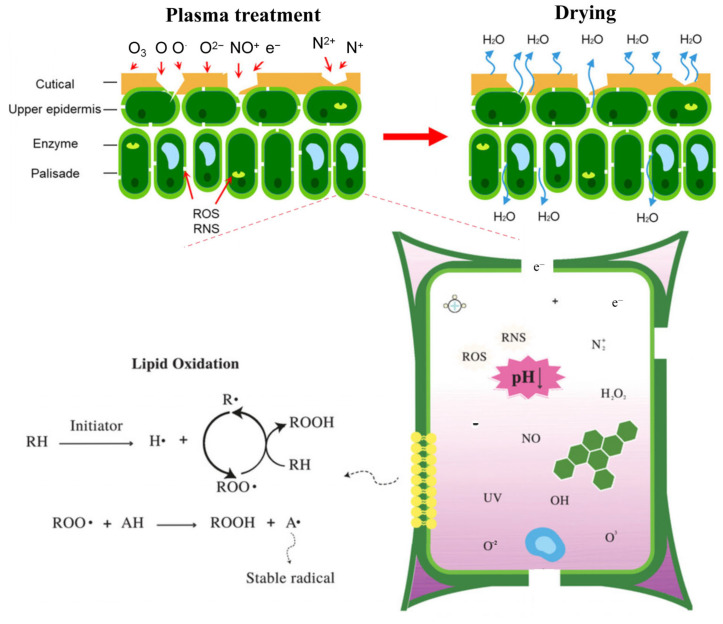
Mechanism of plasma pretreatment and its effect in the drying process [75,112] (reprinted with permission from ref. [112], copyright © 1999–2025 John Wiley & Sons). The dash lines indicate that the part shown is a magnifies image of cells during the CP treatment.

ACP enhances drying performance through synergistic mechanisms (Figure 5). Surface modification via the etching effect from ACP-generated photons, electrons, and ROS/RNS [175], which decomposes surface wax layers and keratin matrices, enhancing hydrophilicity to improve wettability. Surface microcracks increase surface area, facilitating heat transfer and moisture migration [112]. Namjoo et al. [182] observed that ACP treatment significantly improved effective moisture diffusivity in cumin seeds with shortening drying time, attributing this acceleration to plasma-induced surface morphology changes that enhanced internal-to-surface moisture transport. Furthermore, ROS may disrupt hydrogen bonds in water, lowering evaporation enthalpy to promote dehydration. Additionally, cellular structure alteration occurs via chemical bond cleavage, weakening cell walls to form micropores and enlarge intercellular spaces [176]. Karim et al. [183] documented that ACP caused disordered cell arrangement, cellular shrinkage, wall rupture, and significant formation of intercellular voids in fresh plant tissues.

Concurrently, ACP affects dried product quality by inhibiting enzymes (e.g., polyphenol oxidase, peroxidase) through ROS/RNS-mediated modifications, including amino acid side-chain hydroxylation, dehydrogenation, nitration, dimerization, peptide bond cleavage, and disulfide bond formation/rupture, collectively altering enzyme conformation. For metalloenzymes, metal ion oxidation also contributes to inactivation [112]. Processing parameters and plant characteristics (morphology, composition, surface features, moisture content) significantly influence treatment efficacy [112]. For example, while optimal treatment duration enhances drying kinetics, excessive exposure may cause surface hardening or structural collapse [184]. Obajemihi et al. [185] demonstrated that prolonged PAW treatment at higher voltages exacerbated tomato cell disruption, reducing drying time by 25%. Conversely, increased electrode–sample distance diminishes reactive species concentration, compromising drying improvement.

### 3.7. Germination

Regulating seed germination and vegetative growth is essential for plant development and survival [186,187,188,189]. Reactive species generated by cold plasma treatment may alter the physical, chemical, and biochemical properties of plants, thereby positively influencing seed germination [190]. Nitrogen DBD treatment enhances germination rate and vigor [191], while PAW promotes germination via seed matrix modifications [192], demonstrating that PAW treatment effectively promoted germination, possibly due to changes in the seed matrix. Cold plasma increases seed hydrophilicity, optimizing water uptake, while APPJ-induced micro-etching and enzyme activation facilitate germination [193].

ACP treatment of seeds generally involves direct plasma exposure or plasma-activated solutions [190], with germination efficacy influenced by plant species and treatment parameters, the latter including treatment duration, gas composition, and moisture content. Multiple intermittent treatments significantly improved germination potential while slightly increasing germination rate [194], and PAW irrigation significantly enhancing seedling growth [195]. Physically, etching creates seed surface micropores, disrupting waxy barriers to improve oxygen/water permeability for radicle emergence [189]. Biochemically, ROS/RNS act as signaling molecules initiating germination cascades [196], upregulating GA3 oxidase genes to increase GA levels and α-amylase activity while suppressing ABA signaling to break seed dormancy [197,198], activating antioxidant enzymes to scavenge radicals, and accelerating storage compound hydrolysis [190].

### 3.8. Food Packaging Applications

Beyond direct food treatment, ACP is emerging as a critical enabling technology in food packaging. Its application extends from simple surface sterilization to the sophisticated modification of packaging materials to enhance their functional properties. Most packaging materials used in the food industry, such as polyethylene terephthalate (PET), cannot withstand heat, making non-thermal ACP an ideal sterilization method [199]. For instance, studies on packaged cabbage slices, Korean rice cakes, and fish cakes demonstrated significant reductions in bacterial loads, including *Salmonella* spp., confirming the efficacy of this approach [199]. More profoundly, ACP is used to modify the physicochemical properties of polymer surfaces. By generating a rich source of reactive species, UV light, and energetic ions, ACP can induce surface etching and introduce polar functional groups (e.g., hydroxyl, carboxyl). This process increases surface energy, roughness, and hydrophilicity [199]. Enhanced surface polarity and roughness significantly improve the adhesion of antimicrobial or antioxidant coatings to otherwise inert polymer films. This allows for the development of more effective and stable active packaging systems that can slowly release protective compounds onto the food surface, extending shelf-life and improving safety [200]. In addition, ACP can be used to treat surfaces for the immobilization of sensors or indicators. For example, a pH-responsive indicator film was developed using purple basil anthocyanins and nano-TiO_2_. The incorporation of TiO_2_, which can be enhanced by plasma treatment, provided antibacterial activity and improved the film’s mechanical and barrier properties, allowing it to detect food spoilage via a distinct color change [200].

## 4. Cold Plasma Effects on Plant-Based Food Quality

**Figure 6:**
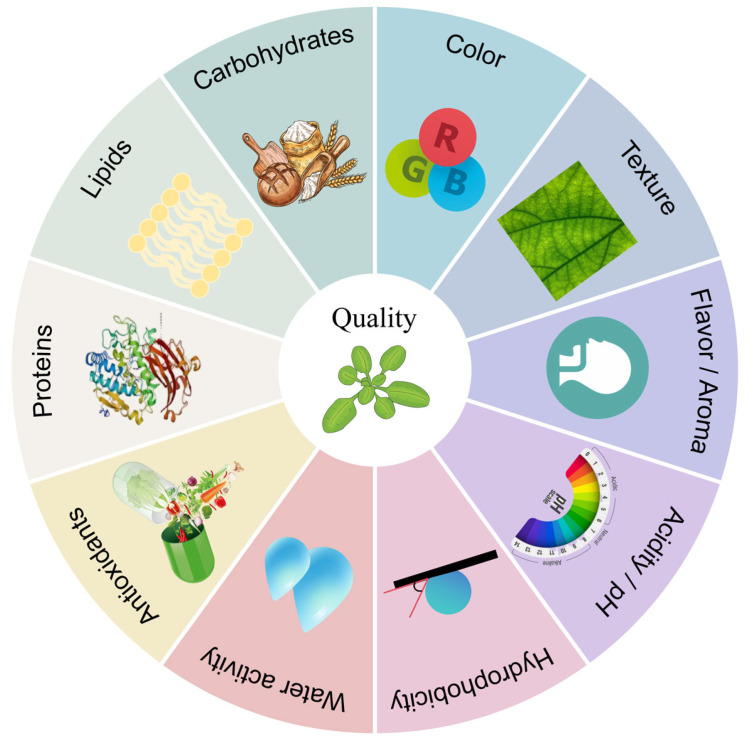
Cold plasma effects on plant-based food quality.

The quality of plant-based foods, defined by sensory attributes, nutritional characteristics, and chemical properties (pH, acidity), critically determines consumer acceptance [201]. Much of the current research on ACP has focused on plant-based raw materials such as grains, nuts, spices, and fresh produce, which are key ingredients in the wider food system. Variations in ACP treatment parameters significantly influence the quality of these materials through modification of surface molecular structures (Figure 6). This alters key physicochemical properties [202,203,204], and further affects textural parameters such as hardness, chewiness, and overall mouthfeel. Such functional modifications demonstrate cold plasma’s potential to enhance sensory attributes and overall quality [201]. This section summarizes ACP effects on plant-based food quality including physicochemical properties, nutritional composition, and functional characteristics (Table S2).

### 4.1. Sensory Attributes and Physicochemical Properties

#### 4.1.1. Color

Color serves as a vital indicator of plant quality, freshness, and nutritional content, directly impacting consumer perception [205]. ACP treatment can induce changes in color, and these effects can be bidirectional—either desirable (e.g., color stabilization, enhanced vibrancy) or undesirable (e.g., browning, bleaching). These changes stem from processing or intrinsic chemical reactions [206,207,208] and the interactions involve oxidation, degradation, or structural modifications (e.g., chain shortening/elongation, hydrogenation, isomerization) [209]. Concomitant cell wall disruption and pH decline from plasma–surface interactions further impact pigment stability [210,211,212]. Studies consistently report measurable color alterations. Examples include darkening in walnut kernels [78], lightening in pistachios [129], enhanced vibrancy in yam powder [213], and improved whiteness in taro starch [214], quantified through parameters like color difference, lightness, and browning index. However, significant variability exists, with several studies reporting no statistically significant color changes in black pepper [79], red pepper powder [215], and *Agaricus bisporus* [86] after plasma treatment. This variability is fundamentally matrix- and parameter-dependent.

The key factors determining the direction and magnitude of change include (i) pigment type and stability, where susceptibility varies significantly between chlorophyll, carotenoids, anthocyanins, and betalains [209]; (ii) food matrix composition, as lipid content, water activity, surface morphology, and the presence of protective compounds critically modulate plasma–pigment interactions [78,129]; (iii) processing parameters, where treatment time, power input, gas composition, and plasma source dictate the balance between pigment degradation, stabilization, or minimal impact, with shorter times/lower power often mitigating changes observed under prolonged exposure [78,86,129,215]. Therefore, systematic investigation into the precise reaction kinetics and degradation pathways of specific pigments under defined plasma conditions is necessary to elucidate fundamental mechanisms. Furthermore, the role of the food matrix requires comprehensive exploration, particularly concerning lipid content, microstructure, and protective components as either a barrier or facilitator of plasma–pigment interactions. Additionally, establishing threshold plasma parameters that trigger significant color changes across diverse food categories is essential for process optimization.

#### 4.1.2. Texture

Texture critically influences plant-based food quality and consumer satisfaction. ACP treatment generally impacts texture, with a primary beneficial effect being the delay in fruit softening (e.g., olives, tomatoes) [201,208,216,217,218,219], potentially linked to plasma’s positive influence on intercellular adhesion and cell wall structural integrity through modification of pectins or cross-linking [220]. However, surface etching and modification are common mechanisms, often leading to observable microstructural changes such as erosion, agglomeration, cracking, and wrinkling [91,92,213,215]. Plasma treatment has been shown to maintain firmness or elasticity in some fruits [216,217,218,219], cause surface erosion or agglomeration in powders while improving functional properties [213], induce cracks on pepper surfaces [215], wrinkle saffron stigmas [92], reduce ion leakage in basil [221], and alter surface morphology in fenugreek seeds [166]. Surface smoothing and increased porosity have also been documented [214,222]. Conversely, several studies report negligible texture alterations, including unchanged hardness in walnut kernels [78]; no significant changes in cinnamon, black pepper, or fennel [85]; and unaltered surface morphology in green tea powder [103]. Outcome divergence stems from the following factors: (i) matrix properties (such as tissue density, cell wall composition, turgor pressure, surface characteristics, moisture) governing plasma penetration depth [85,103,213,220]; (ii) processing parameters controlling energy deposition; and (iii) mechanistic balance between beneficial polymer cross-linking and detrimental degradation or cell rupture [220,221,223,224,225,226]. Consequently, elucidating plasma-induced modifications of key cell wall polymers and their direct relationships with macroscopic texture, combined with systematic investigation of plasma penetration mechanisms (including gas composition effects), is critical.

#### 4.1.3. Flavor and Aroma

Flavor constitutes the perceptual experience elicited by active volatile compounds during mastication [227,228,229,230]. ACP treatment can alter the flavor and aroma profile of plant-based foods through interactions between plasma-generated reactive species (ROS/RONS, UV photons) and volatile organic compounds (VOCs) or their precursors via oxidation, degradation, or formation of new compounds [231]. Studies demonstrate clear alterations, including changes in essential oil composition of turmeric [91]; increases in esters, alcohols, ketones, and fatty acids in edible roses [94]; reduced taste or aroma intensity in saffron [92]; and degraded aromatic quality in ginger [222], with sensory evaluations confirming perceptible differences [215]. However, other research indicates minimal impact, reporting no significant changes in flavor, sensory quality, or volatiles in black pepper [84], cinnamon, fennel [85], or almonds [104]. The presence and extent of flavor modification depend critically on the volatile compound profile and stability, food matrix properties, and processing parameters. The chemical nature of key aroma compounds determines reactivity and profile complexity influences perceived change [91,94,232,233,234,235]. Processing parameters is also paramount. Low doses may only affect surface volatiles or highly reactive compounds, while high doses can penetrate and degrade core volatiles [92,104,215]. Plasma source and gas composition dictate the reactive species generated and their selectivity for different VOCs. In addition, powdered and whole foods exhibit different susceptibilities [84,85,104]. Therefore, investigation is essential, which includes mapping degradation kinetics and the pathways of critical aroma compounds, exploring the role of the matrix (especially lipid content and microstructure in shielding or promoting reactions), and optimizing plasma parameters for specific applications to minimize flavor degradation.

#### 4.1.4. Acidity and pH

Acidity and pH are precisely controlled quality indicators in most processed products. ACP treatment commonly induces a decrease in pH and can alter titratable acidity, primarily attributed to the formation of acidic compounds (e.g., HNO_3_, HNO_2_, H_2_O_2_, H_2_CO_3_) from plasma interactions with atmospheric gases (N_2_, O_2_, CO_2_) and surface moisture, with dissolution of acidic species being the key mechanism [201,231]. Studies report decreasing pH trends in fenugreek extract [166], reductions in pH alongside moisture content [236], and higher titratable acidity in treated winter jujube versus controls [111]. However, some studies found no significant pH change post-treatment, as observed in pistachios [129]. Sample physical state and moisture content strongly influence this variability. Liquids or solids with high surface moisture experience greater dissolution of acidic species, whereas dry powders or waxy-cuticle foods exhibit minimal change, confined primarily to the surface [24,129]. Treatment parameters, which determine reactive species dose and gas composition, are crucial [166,201,231,236]. Additionally, inherent buffering capacity (from organic acids, proteins, and minerals) and initial pH can counteract plasma-induced acidification. Therefore, it is essential to systematically measure the buffering capacity of different plant matrices against this acidification.

#### 4.1.5. Hydrophilicity and Hydrophobicity

Cold plasma modifies plant surface properties through mechanisms like waxy layer oxidation and surface etching, thereby affecting water absorption and retention. These surface alterations may further influence viscosity and rheological behavior in plant-based matrices [201]. Reactive oxygen species (ROS) oxidize C-H or C=C bonds to introduce polar oxygen-containing functional groups such as hydroxyl (-OH), carbonyl (C=O), and carboxyl (-COOH), thereby enhancing hydrophilicity. Such changes are evaluated through water contact angle, surface free energy, and surface chemical composition analysis via X-ray photoelectron spectroscopy or Fourier-transform infrared spectroscopy. Ahmadian et al. [237] found atmospheric cold plasma treatment of *Hyssopus officinalis* preserved its fundamental structure without forming new functional groups, yet significantly reduced the water contact angle, indicating improved wettability. Since surface chemistry and structure jointly influence contact angles in heterogeneous materials [238], increased roughness enhances hydrophilicity independently. This aligns with Shokoohi et al. [239], who demonstrated enhanced hydrophilicity in thyme following atmospheric cold plasma treatment.

#### 4.1.6. Water Activity

Water activity (aw) is a critical indicator of free water availability in materials, influencing microbial growth, enzymatic activity, and chemical reaction rates. Microbial proliferation occurs readily when aw > 0.85 but is inhibited below aw < 0.6. Current research [215] shows higher sample aw correlates with more pronounced microbial inactivation by cold plasma treatment. Common evaluation metrics include moisture content, weight loss rate, and electrolyte leakage rate. When plasma interacts with water molecules, the generated reactive ROS initiate various chemical pathways, thereby reducing the proportion of free water by inactivating microorganisms and inhibiting their metabolic water production [29]. Wiktor et al. [83] observed reduced aw and increased dry matter in black pepper, allspice, and juniper berries following plasma treatment. Similarly, Ramkumar et al. [236] reported decreasing moisture content trends.

### 4.2. Nutritional Value

#### 4.2.1. Antioxidants

Antioxidants inhibit free radicals and prevent oxidation, crucial for plant-based food quality [156,240,241]. Common types include phenolics, vitamins C/E, and flavonoids, evaluated through assays such as DPPH, ORAC, ABTS, and FRAP [231]. Phenolic compounds are key determinants of antioxidant potential [156,231,242]. Cold plasma acts as an abiotic elicitor that may enhance phenolic biosynthesis potentially through ATP induction and accelerated sugar utilization [243], or facilitate phenolic release via cell membrane or wall degradation [244]. However, the effect of cold plasma on total phenolic content remains controversial, as plasma-generated species can degrade phenolic aromatic rings, reducing total phenolics [231]. Flavonoids exhibit similar variability, with increased content observed in *Nelumbo nucifera* powder due to CP treatment, which caused structural changes that facilitate cellular component leaching [231,245]. Similar enhancements for flavonoids post-treatment occurred in black pepper [83], rose [94], and turmeric powder [246]. Vitamin sensitivity varies significantly, with thiamine, folate, and vitamins A/C/E being particularly labile [24,231]. Most research focuses on ascorbic acid stability and indicates minimal impact of cold plasma on ascorbic acid content [24,215]. Bai et al. [204] found no significant change in total phenolics or antioxidant activity but reported reduced allicin content. However, prolonged treatment may decrease levels via ROS-mediated oxidation [201]. Hemmati [91] observed increased phenolics and flavonoids in turmeric but decreased carotenoids and tocopherols with reduced antioxidant activity. GAD treatment enhanced active components in *Crocus sativus*, including crocin, picrocrocin, and total phenolics with enhanced antioxidant capacity [247].

#### 4.2.2. Proteins

Proteins are essential components significantly contributing to nutritional value and quality attributes [248,249]. ACP treatment can modify protein structure and functionality, with plasma-generated ROS/RONS inducing amino acid side-chain oxidation, peptide bond cleavage, and protein cross-linking, altering secondary/tertiary structures and affecting solubility, aggregation, and functional properties [29,60,61,250,251,252,253,254]. Studies report altered amino acid profiles and depolymerization in yam flour [213], and increased content in camellia seeds, possibly due to enhanced extractability [157]. However, inconsistencies exist, as evidenced by preserved total protein content in mushrooms [86]. The extent of modification fundamentally depends on protein structural features along with susceptible amino acid residues and matrix composition (such as lipids, antioxidants, carbohydrates, pH) [86,213,254]. Critical processing parameters encompass plasma source, gas composition, and treatment intensity, where low doses induce minimal unfolding while high doses provoke severe oxidation or aggregation [250,253]. The balance between oxidative cleavage and oxidative cross-linking is modulated by protein type, concentration, microenvironment, and specific ROS/RNS profiles [250,251,252]. Understanding functional property changes in plant protein isolates or concentrates induced by plasma gas-specific modifications requires further investigation. Systematic establishment of dose-response relationships for structural and functional alterations across key plant proteins remains essential.

#### 4.2.3. Lipids

Lipids are hydrophobic or amphiphilic biomolecules crucial for energy storage, signaling, and metabolic regulation [67]. Fatty acids are classified as saturated (SFA), monounsaturated (MUFA), or polyunsaturated (PUFA), with PUFA being prone to oxidative rancidity [255]. ROS targets the positions of unsaturated fatty acids, forming carbon-centered radicals which produce carbon-centered radicals. These initiate lipid peroxidation chain reactions, ultimately generating aldehydes [249] or mediating cross-linking reactions with biomolecules [29]. Plasma treatment generally negatively impacts lipid stability. Zeraatpisheh et al. [129] reported increased UFAs and elevated peroxide value (PV) post-treatment. It is speculated that it may be related to the oxidation of active substances such as hydroxyl radicals, ROS, and singlet oxygen. Thus, adding antioxidants is recommended to mitigate plasma-induced lipid oxidation [201,249]. However, some studies show divergent results, and susceptibility variability stems from lipid composition and location, food matrix properties, and processing parameters. Shirani et al. [104] found no significant PV changes in APPJ-treated almond slices, and Zhang et al. [115] observed minimal effects on fatty acid values in ACP-treated wheat. Esmaeili et al. [126] detected no significant differences in PV or free fatty acid content pre- and post-treatment.

#### 4.2.4. Carbohydrates

Carbohydrates, as primary energy sources in foods, undergo structural modifications under ACP treatment, altering plant-derived food properties [256,257]. ACP induces novel functional groups and conformational changes in polysaccharides, affecting their content and extraction efficiency [160,258]. Starch is particularly susceptible. For instance, plasma-generated RONS and high-energy electrons oxidize glycosidic bonds, degrading starch molecules. This hydrolyzes amylose into hydrophilic monosaccharides while amylopectin breakdown elevates relative amylose content. Concurrently, generated acidic functional groups reduce starch crystallinity, amylose content, gelatinization temperature, swelling power, and viscosity [259]. Polymerization and depolymerization reactions may be closely related to treatment parameters and starch variety [259]. Among these, oxygen and nitrogen plasmas are more likely to induce depolymerization, whereas hydrogen and air plasmas exhibit relatively weaker effects. Resultant structural changes manifest in hydration properties like swelling power, water absorption index, and water solubility index [231]. Zhang et al. [123] noted unchanged wheat gluten but reduced starch content with prolonged plasma treatment. Yang et al. [213] observed increased swelling, solubility, and gelatinization viscosity in yam starch due to oxidation-induced cross-linking or hydrogen bond disruption. Gupta et al. [214] reported altered starch granule morphology, reduced agglomeration, decreased crystallinity, and improved freeze–thaw stability, confirming plasma’s efficacy in modifying starch properties.

## 5. Future Perspectives

ACP technology leverages non-thermal equilibrium to generate reactive species under mild conditions, offering high integration and energy efficiency without denaturing thermosensitive components [260]. Its core mechanism for pathogenic microorganism inactivity combines physicochemical etching and reactive species penetration to disrupt microbial cellular structures and inhibit metabolite accumulation. Redox reactions efficiently degrade pesticide residues like organophosphates, which establish a “zero secondary pollution” decontamination model that reduces exposure risks and extends plant-based product shelf life. As a novel pretreatment technology, ACP optimizes plant drying kinetics, enhances bioactive compound extraction, and improves seed germination and growth [30]. Additionally, ACP holds significant potential to enable personalized nutrition trends. Specifically, it can reduce protein allergenicity (e.g., in soy or peanuts) [256], increase resistant starch for low-glycemic foods, and enhance mineral bioavailability by degrading antinutritional factors [261]. This positions ACP as a foundational technology for tailored health-oriented food systems [262]. Treated samples could retain physicochemical properties, sensory quality, and nutritional value, with some even achieving simultaneous quality preservation and functional enhancement.

Despite these advantages, industrial application still faces bottlenecks: limited action space dimension, incomplete mechanistic understanding due to complex plasma–substrate interactions, and insufficient toxicogenomic assessment of contaminant degradation intermediates [30]. Scaling challenges include plasma source coupling efficiency discrepancies and treatment non-uniformity. Solutions require modular reactor systems, optimized energy efficiency, and enhanced industrial-scale processing [30]. Furthermore, advanced plasma molecular dynamics with machine learning to predict reactive species kinetics and biomacromolecule interactions will provide robust support for process optimization [259]. Since ACP’s effects on plant-based foods are highly matrix-dependent, an intelligent parameter control system must be established. Additionally, strict safety agreements integrated with photoacoustic detection and multi-stage exhaust modules are essential to limit byproducts and ensure operational safety. Clear industrial guidelines must support rational implementation in food safety and quality assurance [201].

## 6. Conclusions

ACP technology demonstrates significant potential in plant-based food processing through pollutant removal, plant growth promotion, and enhanced bioactive compound extraction. This review systematically summarizes recent advances in ACP technology for plant-based foods, focusing on its mechanisms and impacts on plant material quality attributes. Technology effectively degrades pesticide residues while reducing microorganisms and metabolites via synergistic actions of reactive species and electromagnetic fields. Additionally, ACP optimizes plant drying kinetics, enhances active component extraction efficiency, and promotes seed germination and seedling growth.

However, as an emerging technology, ACP still needs in-depth research. Establishing mathematical models’ correlation treatment parameters with quality effects is crucial for process optimization. Additionally, developing large-scale processing facilities is necessary to overcome key challenges in uniformity control. Moreover, the development of standardized assessment systems encompassing chemical composition, bioactivity, and toxicological safety is essential. Formulation of industry application guidelines based on risk–benefit analysis is required to enhance operational efficiency and safety assurance. Addressing these challenges will enable ACP technology to achieve broader industrial adoption while ensuring safety and efficacy.

## Data Availability

No new data were created or analyzed in this study. Data sharing is not applicable to this article.

## Supplementary Materials

The following supporting information can be downloaded at: https://www.mdpi.com/article/10.3390/foods14172999/s1 [78,79,83,84,86,87,91,92,93,94,103,104,123,125,126,129,157,164,166,181,185,204,214,221,237,239,247,263,264,265,266,267], Table S1. Comparative analysis of major ACP generation systems; Table S2. Effects of ACP treatment on the quality of plant-based foods.
