# Supplementary material for: Advances in Atmospheric Cold Plasma Technology for Plant-Based Food Safety, Functionality, and Quality Implications
Source: Foods. 2025 Aug 27;14(17):2999. doi: 10.3390/foods14172999 (PMC12428716; doi:10.3390/foods14172999)
Supplement: Supplementary file 1 [file foods-14-02999-s001.zip › foods-3795682-supplementary.pdf]

Advances in Atmospheric Cold Plasma Technology for Plant-Based Food Safety, Functionality, and Quality Implications

Siyao Liu[a], Danni Yang[a], Jiangqi Huang[b], Huiling Huang[a], Jinyuan Sun[c], Zhen Yang[d]*, Chenguang Zhou[b]*

[a] School of Pharmacy, Jiangsu University, Zhenjiang 212013, China

[b] School of Food and Biological Engineering, Jiangsu University, Zhenjiang 212013, China

[c] Key Laboratory of Geriatric Nutrition and Health, Beijing Technology and Business University, Ministry of Education, Beijing 100048, China

[d] Key Laboratory of Nuclear Agricultural Sciences of Ministry of Agriculture and Zhejiang Province, Institute of Nuclear Agricultural Sciences, Zhejiang University, Hangzhou 310058, China

*Corresponding authors:

Zhen Yang, E-mail: zhen.yang@zju.edu.cn;

Chenguang Zhou, E-mail: zhouchenguang@ujs.edu.cn.

Table S1. Comparative analysis of major ACP generation systems

Technology	Operating Principle	Key Advantages	Key Disadvantages/Limitations	Primary Plant-Based Food Applications
**Dielectric Barrier Discharge (DBD)**	Discharge between two electrodes, with at least one covered by a dielectric. Generates uniform, diffuse plasma over large areas.	- Uniform treatment over large, flat surfaces.<br>- Low energy consumption, scalable.<br>- Suitable for in-package treatment.	- Limited penetration for irregular surfaces.<br>- Efficacy is highly distance-dependent.	- Surface decontamination of grains, nuts, powders.<br>- In-package sterilization of fresh produce.<br>- Modification of packaging films.
**Atmospheric Pressure Plasma Jet (APPJ)**	Plasma is generated in a confined space and ejected as a plume into the open atmosphere.	- Remote and localized treatment.<br>- Effective for complex, 3D, and heat-sensitive surfaces.<br>- No vacuum system required.	- Small treatment area per jet (scalability requires arrays).<br>- Non-uniform treatment can occur.<br>- High consumption of expensive noble gases (e.g., He, Ar) often required for stable operation.	- Decontamination of specific spots on fruits/vegetables.<br>- Treatment of delicate materials like spices and herbs.<br>- Enzyme inactivation on fresh-cut produce.
**Corona Discharge (CD)**	Discharge from a sharp, high-voltage electrode to a larger ground electrode, creating a strong, non-uniform electric field.	- Simple and low-cost setup. - Efficient ozone generation.	- Highly non-uniform treatment.<br>- Small effective treatment area.<br>- Risk of arcing and localized thermal damage.	- Air purification in food storage facilities.<br>- Ozone generation for water treatment (used in washing produce).

**Gliding Arc Discharge (GAD)**	Arc initiated at a narrow gap glides along diverging electrodes, driven by gas flow. Transitions between thermal and non-thermal states.	- High energy efficiency and processing rates.<br>- High production of reactive species.<br>- Can treat large volumes of gas or liquids.	- Non-thermal in downstream regions, but arc core is hot (risk of thermal effects).<br>Primarily suited for gas/liquid phase treatment, not direct solid treatment.	- Degradation of mycotoxins/pesticides in gas streams.<br>- Production of Plasma-Activated Water (PAW).<br>- Fuel conversion and pollution control.
**Plasma-Activated Liquid (PAL)**	Indirect treatment where a liquid (e.g., water) is exposed to plasma, becoming rich in long-lived reactive species.	- Storable and transportable (conditionally).<br>- Can treat complex shapes and internal structures.<br>- No direct exposure of food to plasma/UV.	- Efficacy decays over time; requires specific, often cold, storage conditions for stability.<br>- Indirect action may be less potent than direct treatment.	- Washing/disinfection of fresh produce.<br>- Degradation of pesticides on fruit surfaces.<br>- Seed treatment and germination promotion.

Table S2. Effects of ACP treatment on the quality of plant-based foods.

No.	Sample	Application	Treatment Parameters	Quality Evaluation	References
1	*Juglans regia L.*	Sterilization	RF: 20-50 W; 10-20 min	Darkened color, reduced moisture content, no significant change in hardness.	[78]
2	Black peppercorns	Sterilization	DBD: 9.7-10.6 kV; 7.9-22.1 min	No significant color changes.	[79]
3	*Coriandrum sativum*	Sterilization	APPJ: 47 GHz, 549 W, 0-180 s	Samples became drier with darkened color.	[87]
4	*Curcuma longa* var. Suvarna	Sterilization	DBD: 25 kV; 3-7 min	Dull color, disrupted granular structure, significant increase in total phenolic and flavonoid content, reduced antioxidant activity, mixed effects on volatile oil components.	[91]
5	Fenugreek	Extraction	DBD: Air, 80 kV, 30 min	Surface structural damage, reduced pH, increased water absorption rate, swelling index, viscosity, and lower melting enthalpy; no significant changes in molecular or crystalline structures.	[166]
6	*Crocus sativus* L.	Drying	GAD: 50 Hz, 1 kW, 8 kV, 15-60 s	Pores and cracks formed on stigma surfaces; increased crocin, picrocrocin, safranal, total phenolic content, and antioxidant capacity; no significant differences in sensory attributes or color.	[247]

No.	Sample	Application	Treatment Parameters	Quality Evaluation	References
7	Black pepper seeds, allspice berries and juniper berries	Sterilization	MW: Argon, 20 L/min, 2.45 GHz, 600 W, 15–60 s	Minimal color changes, reduced water activity, increased dry matter content, improved antioxidant activity.	[83]
8	*Allium sativum* L. slices	Drying	DBD: 500 Hz, 65 V, 60-120 s	No impact on color or rehydration rate; surface etching and cell wall collapse observed; no significant changes in total phenolics, antioxidant activity, or allicin content.	[204]
9	*Crocus sativus* L.	Sterilization	APPJ: Air/Argon, 1 L/min, 40-100 W; 1-10 min	Slight declines in color, taste, aroma, and antioxidant activity; wrinkled glandular surfaces; increased crocin content, minor reductions in safranal and picrocrocin levels.	[92]
10	Red mini-roses	Sterilization	DBD: 18 kV, 962 Hz, 20 min;<br>GD: 80 kV, 50 kHz, 20 min	No changes in color or bioaccessibility; increased organic acids, phenolics, antioxidant activity, and volatile compounds.	[94]
11	Agaricus bisporus	Sterilization	DBD: 95 kV, 130 Hz, 10 min	Retention of hardness, weight loss rate, moisture content, browning degree, vitamin C, and total protein levels.	[86]

No.	Sample	Application	Treatment Parameters	Quality Evaluation	References
12	*Pistacia vera* L.	Sterilization	DBD: O_2, 25 kHz, 89 W, 15 kV, 60/120 s	No significant changes in moisture content, color components, peroxide value, hardness, or sensory evaluation; no off-flavors generated.	[125]
13	*Agaricus bisporus*	Drying	DBD: 6 kHz, 29 kV, 30-90 s	Improved antioxidant activity, phenolics, vitamin C retention, color preservation, and rehydration rate.	[263]
14	*Crocus sativus* L.	Sterilization	DBD: 56 kHz, 45 W, 3.4 kV, 1-10 min	Cell wall alterations observed; no significant impact on major chemical constituents.	[264]
15	*Pistacia vera L.*	Sterilization	APPJ: Air-Argon (0%-100%), 10-20 kV, 5-15 min	No significant differences in color, taste, moisture content, peroxide value, free fatty acids, total phenolics, or flavonoids.	[126]
16	*Pistacia vera* L.	Mycotoxin degradation	RF; 12.56 kHz, 80 W, 10/15 min	Lightened color, reduced moisture content; no changes in pH or antioxidant activity; decreased total phenolics and flavonoids; increased unsaturated fatty acids.	[129]
17	Wheat	Mycotoxin degradation	DBD; 60-240 Hz, 60-140 V, duty cycle 20-99%, 25 min	Minimal impact on color, hardness, fatty acid value, or wet gluten content; reduced moisture, protein, and starch levels; no significant differences in fatty acid value or wet gluten content; no damage to color or hardness.	[123]

No.	Sample	Application	Treatment Parameters	Quality Evaluation	References
18	Black pepper	Sterilization	APPJ: 40-60 L/min; 800-1000 W; 0–10 min	No significant changes in color, flavor, or bioactive compounds.	[84]
19	*Hyssopus officinalis* L.	Extraction	DBD: Air/N_2, 5 SLM, 345 Hz, 30 kV, 5-15 min	Reduced surface contact angle, enhanced hydrophilicity, increased total phenolics.	[237]
20	*Centella asiatica* leaves	Drying	PAW: 50 Hz, 30 kV, 15 min	Altered surface cell structure; retained highest levels of phenolics, flavonoids, and antioxidant activity.	[265]
21	*Thymus vulgaris* L.	Extraction	DBD: 17-23 kV, 30-300 s	Minimal color changes; ruptured glandular trichomes; increased hydrophilicity; thymol and linalool levels slightly elevated but not significantly affected.	[239]
22	*Ocimum basilicum* L. cv. Genovese Gigante	Enzyme activity	DBD: 50 kHz, 15/20 kV, 10-30 min	Reduced ion leakage rate; increased moisture content, chlorophyll, carotenoids, antioxidant activity, total flavonoids, and peroxidase activity.	[221]
23	*Colocasia esculenta*	Quality	DBD: 30-34 kV; 2-8 min	Improved surface morphology and roughness; reduced agglomeration; decreased relative crystallinity; enhanced color,	[214]

No.	Sample	Application	Treatment Parameters	Quality Evaluation	References
				whiteness index, aging transparency, freeze-thaw stability, and solubility.	
24	*Cordyceps militaris* (blended *C.militaris*)	Enzyme activity	APPJ: 25 kHz, 12 kV, 30 mA, 30–120 s	Increased fresh/dry weight, cordycepin content, total phenolics, flavonoids, and antioxidant activity.	[266]
25	*Crocus sativus* L.	Sterilization	RF: O_2, 70-110 W; 5-30 min	Elevated color parameters, darker stigma color; reduced picrocrocin, safranal, and crocin content, but quality remained compliant with ISO standards.	[93]
26	*Ziziphus jujuba* Mill.	Drying	PAW: 40 kHz, 650 W, 5 kV, 15-60 s	Increased total phenolics and antioxidant activity; reduced 5-hydroxymethylfurfural.	[267]
27	*Camellia sinensis var. sinensis*	Sterilization	DBD: 20-25 kV; 2-8 min	Unaltered surface morphology; well-preserved total phenolics, flavonoids, antioxidant activity, volatile compounds, and sensory quality.	[103]
28	*Camelina sativa* seed	Extraction	DBD: 15-21 kV; 2-16 min	Increased protein content; minimal impact on color parameters; elevated linolenic and linoleic acid levels; reduced palmitic acid content.	[157]

No.	Sample	Application	Treatment Parameters	Quality Evaluation	References
29	Almond slices	Sterilization	APPJ: Helium, 10 SLM, 17 V, 2.26 A, 5-20 min	No changes in color, peroxide value, or sensory attributes; acceptable hardness variations.	[104]
30	*Lycium barbarum* L.	Drying	DBD: 20 kHz, 750 W, 15-60 s	Increased L, *a*, b* values; reduced ΔE; improved rehydration rate; chemical content initially rose then declined with prolonged treatment; microstructural changes favored extraction and drying.	[181]
31	*Syzygium aromaticum*	Extraction	DBD: N_2, 1 L/min, 345 Hz, 15 kV, 5-15 min	Substantial microstructural rupture; color changes; increased total phenolics and antioxidant activity.	[164]
32	*Solanum lycopersicum* L.	Drying	DBD: 1 kHz, 50/70 V, 2-10 min	Improved appearance, rehydration, porosity, phenolics, flavonoids, soluble solids, lycopene, and ascorbic acid; reduced chewiness, gumminess, and hardness.	[185]
